# Visible-Light-Driven Reduced Graphite Oxide as a Metal-Free Catalyst for Degradation of Colored Wastewater

**DOI:** 10.3390/nano12030374

**Published:** 2022-01-24

**Authors:** Mahmoud Mazarji, Niyaz Mohammad Mahmoodi, Gholamreza Nabi Bidhendi, Tatiana Minkina, Svetlana Sushkova, Saglara Mandzhieva, Tatiana Bauer, Alexander Soldatov

**Affiliations:** 1 Academy of Biology and Biotechnology Named D.I. Ivanovsky, Southern Federal University, 344090 Rostov-on-Don, Russia; minkina@sfedu.ru (T.M.); snsushkova@sfedu.ru (S.S.); msaglara@sfedu.ru (S.M.); bauer@sfedu.ru (T.B.); soldatov@sfedu.ru (A.S.); 2 Department of Environmental Research, Institute for Color Science and Technology, Tehran 1668836471, Iran; mahmoodi@icrc.ac.ir; 3 School of Environment, College of Engineering, University of Tehran, Tehran 1417614411, Iran; ghhedni@ut.ac.ir

**Keywords:** reduced graphite oxide, preparation, characterization, photocatalysis, Fenton, dye degradation, visible-light-driven catalyst

## Abstract

Reduced graphite oxide (rGO)-based materials have demonstrated promising potential for advanced oxidation processes. Along with its distinctive 2D characteristics, rGO offers the prospect of catalytic degradation of various kinds of organic pollutants from aqueous environments. The practical application of rGO as a metal-free catalyst material to promote the Fenton reaction depends on the degree of rGO reduction. In this regard, the rGO was prepared according to oxidation by modified Hummers’ method and two-step reduction via hydrothermal and calcination in the N_2_ atmosphere. The as-prepared rGO was characterized in terms of X-ray diffraction, Fourier-transform infrared spectroscopy, thermal gravimetric analysis, scanning electron microscopy, UV-vis absorption spectroscopy, and transmission electron microscopy. The effectiveness of as-prepared rGO as a photocatalyst and the metal-free catalyst to decolorize different textile dyes, including basic red 46, basic red 18, and methylene blue, was investigated in visible/rGO and visible/rGO/H_2_O_2_ systems. The impact of operational factors such as catalyst dose, pH, and initial dye concentration was examined. The dye degradation process was investigated by the pseudo-first-order kinetic model. In addition, the recyclability of rGO in the visible/rGO/H_2_O_2_ system was examined.

## 1. Introduction

The variety of organic dyes produced annually in different industries such as textile, paper, and plastic have been estimated to account for more than 450,000 tons globally [1]. The fact that many of these dyes are constantly discharged into the environment as effluent throughout the manufacturing and application processes is an unpleasant side effect of their widespread usage, because their complex and synthetic origins are extremely hazardous, possibly carcinogenic, and mutagenic, posing a serious threat to human health and disrupting the ecosystem of receiving waterways [2,3,4,5,6,7]. Traditional water and wastewater treatment practices are inadequate to meet the difficulties associated with textile dyes [7,8]. The advances made by nanomaterials hold the promise of evolving traditional water and wastewater treatment technology to deal with environmental issues related to textile effluents [9,10,11,12]. Among these, photocatalytic and catalytic oxidation is well-known to be a promising and green technology in the field of advanced oxidation processes with high efficiency [13,14,15,16,17]. This process has been extensively investigated to mineralize a wide range of organic pollutants, including dyes, to relative nontoxic end products, i.e., water and carbon dioxide [9,11,18,19,20,21].

The unique properties of graphene, such as the large theoretical specific surface area, tunable bandgap, outstanding electron mobility, exceptional thermal conductivity, and robust mechanical strength, offer tremendous opportunities in advanced oxidation processes [5,22,23,24,25,26,27]. In this context, graphene and related materials have been used as a new class of carbocatalysts in a standalone mode [28,29,30,31,32]. Based on the literature review, variation of the ratio of sp^2^ to sp^3^ fractions can transform graphite oxide (GO) from an insulator to reduced graphite oxide (rGO) as a conductive material due to the restoration of some of the sp^2^ domains [33,34,35,36,37]. Reduced graphite oxide, largely sp^2^ hybridized material, possesses superior electron conductivity, resulting in retardation of the recombination of electron and hole pairs during photoactivity [38]. The reduction method with the minimum introduction of defects on the graphene framework is desired in order to enhance photocatalytic performance [34,35]. The progress on the usage of the oxidative and reductive form of graphene as an independent semiconductor material has perhaps received less coverage than is warranted.

In traditional Fenton reactions, the redox cycle of dissolved Fe(II)/Fe(III) species produces powerfully oxidizing hydroxyl radicals (HO^•^) [39]. For the treatment of hazardous and refractory dye contaminants, heterogeneous Fenton processes are considered one of the most promising advanced oxidation methods [40], and this method has been widely used to degrade textile effluent [41]. Despite all the success, its application is still limited by iron sludge formation due to Fe(III) precipitation [42]. In light of this situation, rGO, a metal-free Fenton carbocatalyst, provides good potential for modifying the chemical environment of the Fenton reaction. Reduced graphite oxide (rGO) has recently been shown to be a promising metal-free catalyst for promoting the Fenton reaction to degrade phenol [28]. Given that rGO and other members of the graphene family can be used as semiconductors and that the so-called photo-Fenton reaction is an alternative to the Fenton reaction, it is worth expanding on the previous study on the catalytic activity of rGO for dye degradation.

In this study, the two-step post-treatment procedure was used to create rGO in this study with the ability to get activated upon visible light irradiation, and several methods were used to characterize it. The application of as-synthesized rGO in photocatalysis and the metal-free Fenton process was investigated for degradation of several textile dyes, including basic red 46 (BR46), basic red 18 (BR18), and methylene blue (MB). These dyes are often used to give wool, silk, and cellulosic fibers bright hues [43]. These cationic dyes have at least one azo group attached to aromatic rings [44]. Due to coloring, toxicity, mutagenicity, and carcinogenicity characteristics, the treatment of these dyes is of high priority. As a result, BR46, BR18, and MB were chosen as model dye pollutants for further research into the visible-light activity of rGO. To the best of our knowledge, detailed investigations on the catalytic activity of solely rGO in terms of catalyst dosage, initial dye concentration, and pH of solution are still lacking. The effectiveness of rGO in the metal-free Fenton reaction (i.e., visible/rGO/H_2_O_2_ system) was assessed, and the associated mechanism was elaborated.

## 2. Materials and Methods

### 2.1. Materials

Sigma-Aldrich company (Darmstadt, Germany) provided all compounds, which were used without additional purification. For all studies, diluted NaOH and H_2_SO_4_ solutions were employed to keep the pH at the appropriate level. All aqueous solutions were made using water that had been doubly distilled.

### 2.2. Preparation of Graphite Oxide and Reduced Graphite Oxide

Preparation of graphite oxide was accomplished following modified Hummers’ method, and the detailed procedure was documented in one of our previous works [6]. Briefly, in an ice bath, 2 g purely natural graphite and 4 g NaNO_3_ were added into 100 mL H_2_SO_4_ 98% solution, and this solution was stirred vigorously. Thereafter, 10 g KMnO_4_ was slowly introduced, and the resultant mixture was agitated for two days at room temperature. Deionized water followed by H_2_O_2_ was added to terminate the synthesis. The product was rinsed numerous times with a 5% HCl solution and deionized water during centrifugation. The resulting powder was heated to dryness at 60 °C overnight. Graphite oxide was obtained after probe ultrasonication for one hour.

To maximize the reduction of GO as proposed by the literature [45], a two-step reduction technique was adopted. In the pre-reduction step, the 10 mg/mL aqueous solution of GO at pH of 11 was prepared and then put into a Teflon-lined autoclave. As reported elsewhere, the pH was adjusted to 11, since graphene sheets have been reported to be extremely variable to the pH during the hydrothermal reduction stage [46]. The autoclave was placed at 100 °C for 24 h. The black rGO powder was collected after washing with water several times, followed by drying at 60 °C for 24 h. Lastly, the resulting rGO was calcined in the N_2_ environment for three h at 400 °C.

### 2.3. Characterization

The phase and crystallite size of the sheets were investigated using X-ray diffraction (XRD, Siemens D-5000 diffractometer, Bremen, Germany). Thermal gravimetric analysis (TGA, Perkin-Elmer PYRIS Diamond, Waltham, MA, USA, scanning electron microscopy (SEM, LEO 1455VP scanning microscope, Munich, Germany), Fourier-transform infrared spectroscopy (FTIR, Perkin-Elmer spectrophotometer spectrum one, Waltham, MA, USA), and UV–visible (UV–Vis spectrophotometer Perkin-Elmer Lambda 25, Waltham, MA, USA) were employed to study the thermal behavior, surface morphology, functional groups and optical property of the prepared materials, respectively. Moreover, transmission electron microscopy (TEM) was used to examine the size of sheets using a Tecnai G2 microscope (FEI, Hillsboro, OR, USA).

### 2.4. Dye Degradation Experiments

The model dyes, including basic red 46, BR18, and MB, were chosen to investigate the effectiveness of rGO under different conditions. The visible light source was 150 W with a 420 nm cut-off filter (OSRAM, Munich, Germany). The used lamp and photoreactor schematic were described in previously published papers [47,48]. Briefly, the distance from the lamp to the top of the solution was 15 cm. Experiments were carried out at a temperature of 26 °C, and thanks to effective air exchange provided by an external cooling fan, the samples never increased in temperature by more than 5 °C during the course of the treatment. An aquarium air pump was used to assure the saturation of dissolved oxygen in the reactor. In a typical procedure, a 500 mL solution containing varying concentrations of rGO (5–20 mg) and dye (20 mg/L) was organized. The adsorption–desorption equilibrium was achieved in the darkness between the rGO and dye molecules.

After turning the light on, a 3 mL sample solution was extracted at frequent intervals, and its effluent was centrifuged. The change in maximum absorbance of dyes at wavelengths of 531, 488, and 662 nm for BR46, BR18, and MB, respectively, were used to monitor the process. According to Beer Lambert’s law, the normalized maximum absorbance (A_t_/A_0_) was used as the normalized temporal concentration changes (C_t_/C_0_) [49]. Moreover, the activity of rGO was evaluated by a pseudo-first-order kinetic model [50,51].

Similar to the above procedure, in a metal-free Fenton reaction, 0.04 mM of H_2_O_2_ was added to the suspension containing the optimum amount of rGO. The effects of pH (2–7) and initial dye concentration (20–50 mg/L) were evaluated in order to determine the optimum amount of rGO and optimum pH of the solution, respectively. The mineralization activity was investigated using a total organic carbon (TOC) analyzer (Shimadzu, TOC-L, Tokyo, Japan). All experiments were conducted at room temperature.

### 2.5. The Recyclability and Stability Test of Reduced Graphite Oxide

For the two following cycles, the recyclability and stability of rGO were investigated during the degradation of BR46 under optimal conditions. The BR46 solution was refreshed for the next run to assess the capabilities of the restored rGO under identical conditions. When the first cycle was over, the spent catalyst was recycled and characterized in terms of SEM.

## 3. Results and Discussion

### 3.1. Characterization

**Figure 1:**
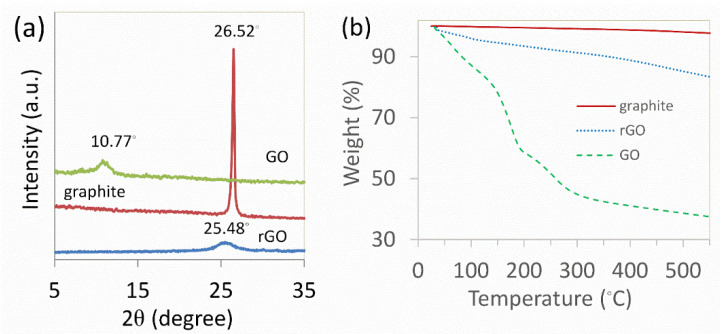
(**a**) XRD and (**b**) TGA spectra of graphite, GO, and rGO.

X-ray diffraction was used to characterize the crystal structures of graphite, GO, and rGO, as shown in Figure 1a. Graphite’s feature diffraction peak showed a very acute and powerful increase at 26.52° (002), corresponding to d-spacing of 0.336 nm. The 002 peak appeared at a lower angle at 10.77° with d-spacing of 0.821 nm in the GO sample after the oxidation and exfoliation. This discovery showed that water molecules and functional groups containing oxygen increased the interlayer gap of the graphitic structure. Following the removal of the functional group of GO under hydrothermal conditions, a 002 peak at 25.48° formed with a d-spacing of 0.349 nm, indicating that the d-spacing of GO was reduced. The XRD measurements results were consistent with prior publications [25,52,53,54]. The assessment of various d values obtained in the XRD experiment demonstrated that, although the d-spacing of rGO by 0.349 nm was significantly lower than that of GO (0.821 nm) and still more than parent graphite (0.336 nm), indicating that the slight number of oxygen functional groups retained after the reduction reaction [23].

Thermal gravimetric measurements were performed in the nitrogen atmosphere. A result of graphite, GO, and rGO is shown in Figure 1b. Graphite demonstrated high thermal stability throughout the test, whereas GO breakdown was shown to proceed in two significant phases. The first stage was connected with the reduction of labile oxygen functional groups, whereas the second was involved with the complete oxidation process [8,25,55]. It was revealed that the weight reduction of rGO was better than that of GO, owing to the elimination of the labile oxygen-containing functional groups. These findings were in agreement with the literature [46,52].

**Figure 2:**
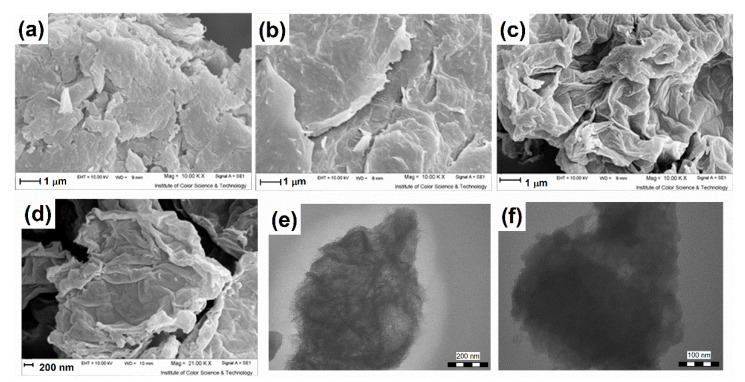
SEM images of (**a**) graphite, (**b**) GO, (**c**,**d**) rGO, and (**e**,**f**) TEM images of rGO.

The morphology of graphite, GO, and rGO is investigated by SEM (Figure 2). As illustrated in Figure 2b, the layer organization in GO was no longer identical to the flat and straight shape of graphite (Figure 2a) owing to substantial interlayer interaction. In particular, following the acidic treatment using Hummers’ approach, the edge and surface of GO were wrinkled and coarsely formed [25,52]. As shown from both high- and low-magnification images in Figure 2c,d, rGO sheets were corrugated and crumpled shapes. These two characteristics were inherent to the nature of rGO sheets, while its two-dimensional structure became thermodynamically stable through bending. Similar to former reports for rGO, aggregation of crumpled graphene sheets was observed elsewhere [56,57,58]. The TEM images of rGO in different magnifications are shown in Figure 2e,f. The macrosized planar sheets are clearly visible, showing that the graphene morphology’s high surface/volume ratio and two-dimensional structure are well preserved [59,60]. Furthermore, as shown in Figure 2e, rGO contained a lot of corrugations and scrolling.

**Figure 3:**
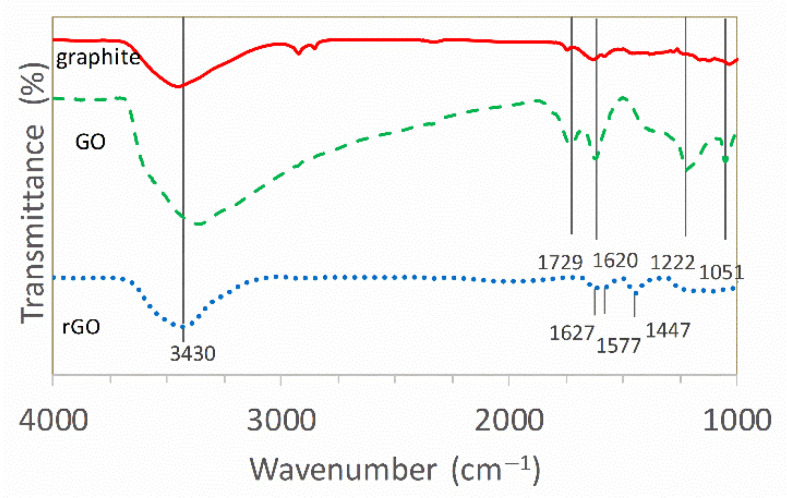
The FTIR spectra of graphite, GO, and rGO.

Figure 3 depicts the FTIR spectra of graphite, GO, and rGO. The existence of the OH stretching, attributable to surface OH groups and adsorbed water between graphene interlayers, was revealed by the band at a wavenumber of 3360 cm^−1^ for GO (3430 cm^−1^ for rGO and 3445 cm^−1^ for graphite). More crucially, as a consequence of the massive load of oxygen-containing functional groups produced by the oxidative process, the peak of the OH group in the GO sample became more apparent than that of others. Major stretching vibrations were observed in graphite oxide and graphite at 1729, 1620, 1222, and 1051 cm^−1^, which corresponded to –C=O stretching (COOH group), C=C, C–O, and C–O–C groups, respectively [6]. The presence of C–O–C bonds is in very good agreement with the highly accurate synchrotron-based measurement of X-ray absorption near the edge [61]. The presence of the C=C ring stretching at 1577 cm^−1^ resulted from the rGO’s skeleton vibration [8,62]. Furthermore, the peak at 1627 cm^−1^ was assigned to the vibrations of adsorbed water and the skeleton vibrations of unoxidized graphitic domains [8]. In comparison to graphite and GO samples, the intensities of oxygen-containing functional groups’ bands were observed to weaken strongly in the rGO sample.

**Figure 4:**
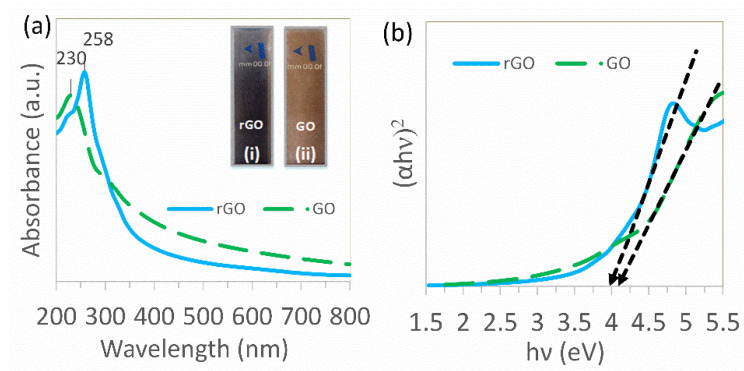
(**a**) Optical absorption spectra of GO and rGO (the inset is a photograph of dispersion of (i) rGO and (ii) GO in water). (**b**) Optical bandgap spectra for GO and rGO (the Tauc linear fits to the data are shown by the black dotted arrows).

The optical properties of GO and rGO are given in Figure 4a. As can be seen, GO exhibited a high absorption peak around 230 nm and a minor shoulder peak about 303 nm, which developed as a consequence of π-π* transition of the aromatic C–C ring and 𝑛-𝜋* transition of C=O bond. Furthermore, rGO showed an absorption peak at 258 nm that was red-shifted as a result of electrical configuration in graphene sheets during GO reduction [52,63,64,65]. This observation was confirmed by other researchers’ studies, in which the C K near-edge X-ray absorption fine structure (NEXAFS) spectra were used to show the increase in the intensity of the sp^2^-derived unoccupied states π* band in the rGO sample [66]. The inset of Figure 4a presents the color change of water dispersion of GO and rGO from yellow-brown to black, indicating the partial restoration of the conjugation network within the carbon structure [65].

The energy band gap of GO and rGO can be expressed by the following equation for a direct transition [67].

(αhν)^2^ = A(hν − E_g_) (1)

where α, hν, A, and E_g_ are the absorption coefficient, photon energy, constant, and bandgap within this sequence. The plot of (αhν)^2^ vs. hν yields a straight line, the intercept of which on the horizontal axis provides the energy bandgap. Figure 4b shows the reflectance spectrum of GO and rGO (an indirect bandgap) transformed according to Equation (1) plotted vs. the photon energy (also called Tauc plot). The linear trends in Figure 4b were the tangent of the Tauc plot near the maximum slope point where the plot contained a sufficiently linear region [68]. The tangent line (Tauc linear fit) should be constructed in the region, showing a steep, linear increase of light absorption with increasing energy [69]. In the final step of the calculation, the *x*-axis intersection point of the linear fit was used to give an estimate of the bandgap energy [70]. The bandgap of GO was projected to be 4.08 eV, as illustrated in Figure 4b. In comparison, the bandgap of rGO was red-shifted to lower energy (3.97 eV), allowing electron excitation by lower energy. This trend was in agreement with another report [52]. This could be due to losing some oxygen functionality groups and restoring some of its sp^2^ structure [64]. The results from the absorbance spectra experiment indicated that engineering the bandgap of rGO could be achieved under hydrothermal conditions.

### 3.2. Dye Degradation Experiments

#### 3.2.1. Effect of Catalyst Dosage

**Figure 5:**
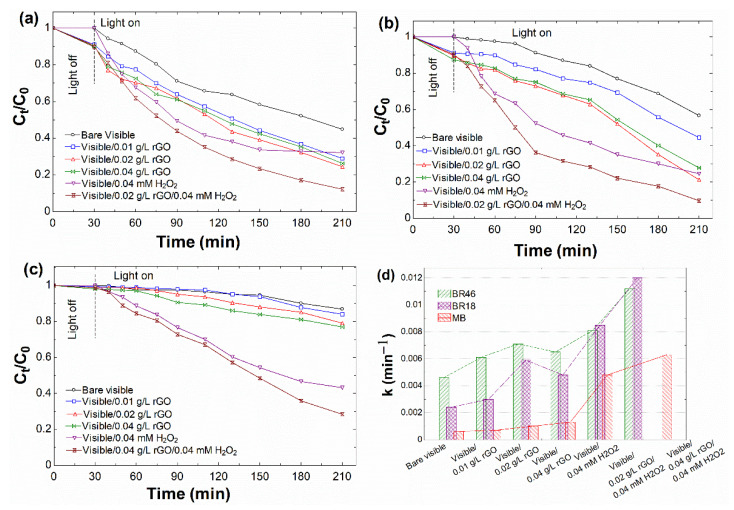
Effect of rGO dosage on photodecomposition of different days with an initial dye concentration of 20 mg/L: (**a**) BR46 (pH 4.65), (**b**) BR18 (pH 4.68), and (**c**) MB (pH 4.58) following the effect of addition of 0.04 mM H_2_O_2_, and (**d**) their apparent rate constants.

The dosage of the catalyst in the dye degradation process is of influential operational parameter [2,71]. Figure 5a–c present the profiles of adsorption and photodegradation of dyes as a function of different rGO dosages (0.01~0.04 g/L). Before the light was switched on, all experiments were conducted following a 30-min physical adsorption period in the dark to establish adsorption and desorption equilibration. When the reaction achieves equilibrium after 30 min in the dark, as shown in Figure 5a for BR46, Figure 5b for BR18, and Figure 5c for MB, the adsorption data for all days showed a similar pattern where the dye concentration was hardly lowered in the presence of rGO, owing to the small quantity of catalyst used. The adsorption might possibly be occurred due to the presence of the marginal amount of oxygen functional groups on the surface of rGO. As a consequence of the oxygen-containing functional groups, the cationic dye molecules are inclined to adsorb onto the surface of rGO by electrostatic interaction [72].

In the photocatalysis step of BR46 and BR18, the increment of degradation could be observed up to 0.02 g/L. However, in the dosage higher than 0.02 g/L, the efficiency of the reaction was found to decrease. It mainly might be due to increased turbidity by excessing black graphene and blocking photons’ penetration in the solution. Another reason for this was the possible aggregation of particles in higher dosage and, consequently, reduction of the active surface area of rGO [35].

On the other hand, as mentioned earlier in the photocatalysis step, this behavior was not found for MB degradation. The addition of catalyst did not significantly improve the degradation efficiency compared to the benchmark, i.e., barely visible. This phenomenon might be attributed to the too many MB dyes adsorbed onto graphene sheets, which would absorb incident light and thus impeding the generation of photo-induced electrons and holes. A similar finding was previously reported for the photodecomposition of MB by graphene-based composites under UV light irradiation [73]. Based on the above experiment, 0.02 g/L for BR46 and BR18 and 0.04 g/L for MB were taken as optimum rGO dosage in further experiments.

The visible/rGO process mechanism has not been discussed well in the literature [63]. Since the bandgap of rGO was 3.97 eV irradiation with light energy higher than its bandgap, the electron and hole pairs will be excited upon irradiation (Equation (2)).

(2) $$\mathrm{rGO}\overset{\mathrm{Visible}}{\to }e^{-}+h^{+}$$

If these electron and hole pairs were effectively separated, they may migrate to rGO and take part in other photo-induced processes. The photogenerated hole in the aqueous phase may just react with adsorbed water molecules or directly oxidize different organic compounds. Simultaneously, the electron in the conduction band may be readily scavenged with O_2_ as the primary acceptor to generate an oxygen superoxide radical anion (O_2_^−•^) [2,21]. It is crucial to note that earlier results indicate that graphene-derived compounds’ valence band edge may not be positive enough just to oxidize water directly [36,37,74]. Therefore, the visible/rGO system demands another route to address the issue associated with the shortage of HO^•^ to compensate for the lack of oxidizing radicals. The well-known ability of rGO in the photocatalytic system is to store and shuttle the photogenerated electrons due to its superior electron conductivity and mobility [75]. With that in mind, this ability can result in the effective charge separation and accumulation of electrons on the sheets, thus transforming the electron along with graphene sheets to react with O_2_ or other sources of electron acceptors to form HO^•^ for the further photocatalytic degradation of dyes [76,77,78].

According to the literature, rGO was a suitable metal-free catalyst to promote the Fenton reaction at acidic pH values [28]. In this context, the sunlight-assisted Fenton-like activity of rGO was tested against degradation of phenol [29]. In other words, the addition of H_2_O_2_ into the visible/rGO system could easily improve the generation of HO^•^ by turning the photocatalytic reaction into a metal-free visible-assisted Fenton-like reaction [79]. In metal-free catalysis (i.e., visible/rGO/H_2_O_2_), H_2_O_2_ is predicted to synergize the photodegradation of organic contaminants by enhancing HO^•^. With that in mind, at the optimum catalyst dosage for each dye, a low amount of H_2_O_2_ (0.04 mM) was supplemented to the system. In the visible/H_2_O_2_ system, due to the photolysis of H_2_O_2_, HO^•^ radicals produce, which act as strong oxidants [19,80]. Compared to the control bare H_2_O_2_, the addition of rGO led to remarkably enhanced efficiency due to synergistic effects among rGO and H_2_O_2_ (Figure 5a–c). In the visible/rGO/H_2_O_2_ system, O_2_ is the recipient of the electron to yield O_2_^−•^, according to Equation (3). As previously mentioned, H_2_O_2_’s role in this system is to compensate for the absence of O_2_ and scavenge the photo-induced electron, according to Equation (4) [18,19,81].

(3) $$\mathrm{rGO}\left(e^{-}\right)+{\;O}_{2}\to O_{2}{}^{-\;∙}$$

(4) $$\mathrm{rGO}\left(e^{-}\right)+{\;H}_{2}O_{2}\to {\mathrm{HO}}^{•}+{\mathrm{OH}}^{-}$$

Furthermore, H_2_O_2_ reacts with O_2_^−•^ to form HO^•^ as given by Equation (5) [18,82]. The photodegradation of used dyes could be achieved through attacks by HO^•^ at their weakest chemical bonds, in accordance with Equation (6) [18].

(5) $$H_{2}O_{2}+{\;O}_{2}{}^{-\;∙}\to {\mathrm{HO}}^{•}+{\mathrm{OH}}^{-}+O_{2}$$

(6) $$\mathrm{Dyes}+{\;\mathrm{HO}}^{•}\to \mathrm{Prdocts}$$

By comparing the apparent pseudo-first-order kinetic rate, further interpretation of the preceding data was gained. Figure 5d shows the constant rates (k) determined from the linear fitting of ln(C_0_/C_t_) versus time. Table 1 summarizes their related correlation coefficients (R^2^) and k values. The order of rate constants in all series testing for BR46 was greatest for the bare visible and varying concentrations of rGO. However, after adding H_2_O_2_, BR18 tended to show the highest decomposition rate. In comparison, 20 mg/L MB was poorly degraded by rGO, bare H_2_O_2_, and rGO/H_2_O_2_. As shown in Table 1, there were very low kinetic rates in the case of experiments without the addition of catalyst (i.e., barely visible), despite the high accuracy of obtained R^2^. This means that the employment of bare visible solely could not afford the photodegradation of dyes, and the presence of catalyst was necessary to obtain faster kinetic rates.

#### 3.2.2. Effect of Initial Dye Concentration

**Figure 6:**
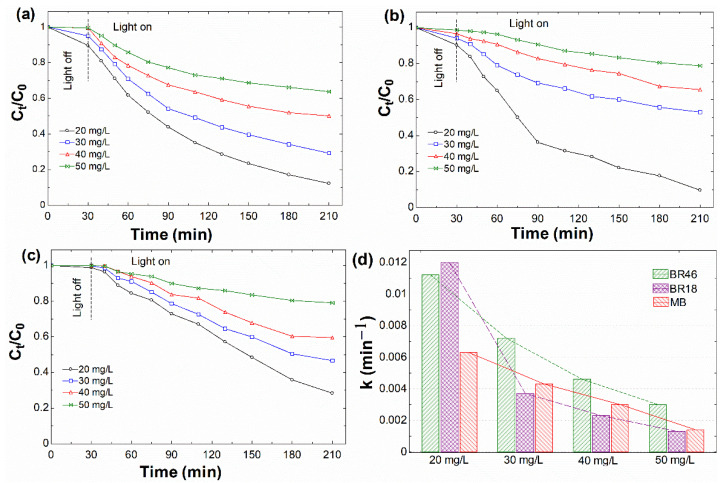
Effect of initial dye concentration on photodecomposition of different basic days at optimum catalyst dosage in visible/rGO/H_2_O_2_ system and pH of 4.5: (**a**) BR46, (**b**) BR18, (**c**) MB, and (**d**) their apparent rate constants.

The dye concentration is the additional key parameter that needs to be considered, as the process’s efficacy is affected tremendously by it [83]. In this context, the effect of variation of initial dye concentration from 20 to 50 mg/L was investigated. At the same time, other parameters were kept constant at their previously optimized conditions. Figure 6a–c exhibit dye degradation dependency on initial dye concentration. It is essential to mention that the adsorption process showed the reliance on the dye concentration as the adsorption decreased lower at the higher dye concentration. When the light was turned on, the change of curves followed a similar trend for all dyes. As the concentration increased, the depletion in normalized concentration value was more pronounced. The resulting data could be rationalized because more organic substances made it impossible for the system to perform well as dye concentration increased. In addition, increasing the dye concentration weakened the pathway for photons incoming into the solution, thus lessening the photonic absorption on the rGO surface and reducing dye degradation efficacy [83].

#### 3.2.3. Effect of pH

**Figure 7:**
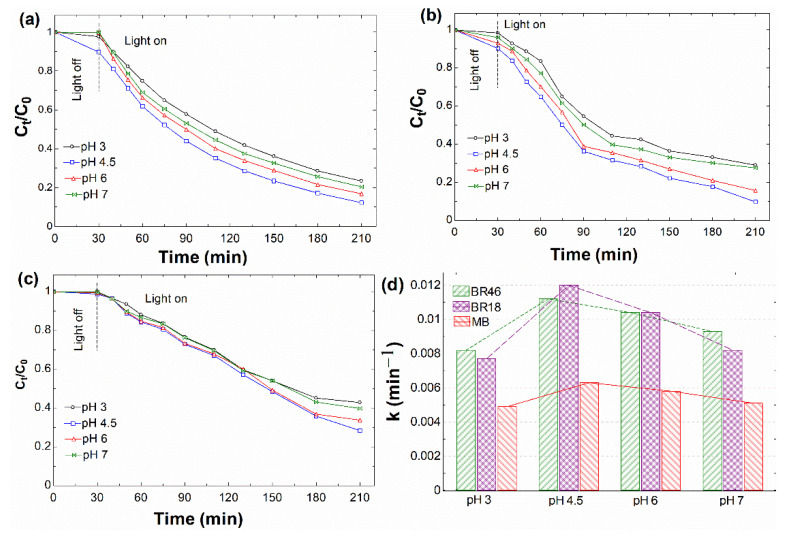
Effect of pH of solution on photodecomposition of different basic days with an initial dye concentration of 20 mg/L at optimum catalyst dosage in visible/rGO/H_2_O_2_ system: (**a**) BR46, (**b**) BR18, (**c**) MB, and (**d**) their apparent rate constants.

The variation pH of the solution can affect the surface charge of the catalyst and its tendency to form aggregations [84]. Extra experiments were carried out to ascertain the effect of pH value in the range from 3 to 7 on the photodecomposition of dyes in the metal-free Fenton reaction (i.e., visible/rGO/H_2_O_2_ system). The results are displayed in Figure 7. For the adsorption step in the dark conditions, as mirrored in Figure 7a–c, by increasing the pH to the value of 4.5, the dye adsorption efficiency became the highest, irrespective of the different dye structures. This might happen because positively charged basic molecules in the solution were neutralized at higher pH values than 4.5. As a result of the neutralization process, the electrostatic forces of attraction between rGO and BR46/BR18/MB were lessened, and with rising pH, gradual absorption of dyes was observed [85]. The analogous activity trend was observed in degradation of all basic dyes, where the C_t_/C_0_ value increased with an increase in pH until it reached the maximum at pH 4.5. With pH higher than 4.5, the normalized concentration decreased with increasing pH. The reason for this observation might be that as the pH became more acidic, a high amount of sedimentation took place due to aggregation and low repulsion within the graphene sheets [86]. Therefore, this fact contributes to hindering the photoactivity of rGO sheets. By increasing the pH, the higher negative charge of rGO would contribute to (i) a lower tendency to form aggregates; hence, an increase in the photoactivity of rGO; and (ii) facilitation of interaction between negatively charged of rGO sheets and the cationic surface of the basic dyes, thus increasing its adsorptive ability. As demonstrated by the calculated kinetic rates in Figure 6d, the efficiency decreased at pH higher than 4.5. It seemed that with basic pH, the impact of rGO in the visible/rGO/H_2_O_2_ system became insignificant compared to the adverse effect of H_2_O_2_. In this condition, H_2_O_2_ as a rate-limiting step could (i) directly break down to water and O_2_ rather than well-known HO^•^ and (ii) indirectly be wasted by hydroperoxy anion (HO_2_^−^) as oxidizing species of H_2_O_2_ in the basic medium [87]:

(7) $$2H_{2}O_{2}\overset{\mathrm{visible}}{\to }2H_{2}O+O_{2}$$

(8) $$2H_{2}O_{2}+{\mathrm{HO}}_{2}^{-}\to H_{2}O+O_{2}+{\mathrm{HO}}^{•}$$

(9) $${\mathrm{HO}}^{•}+{\;\mathrm{HO}}_{2}^{-}\to H_{2}O+O_{2}^{-}$$

Another contributing factor to observing higher activity in this pH value is that it was near to the own pH of dye solution devoid of using any additives, thus avoiding their presence and adverse scavenging effects [2]. From the above results, it could be deduced that the optimum pH value was 4.5 for all the basic dyes.

#### 3.2.4. UV-Vis Spectral Changes of Dyes

**Figure 8:**
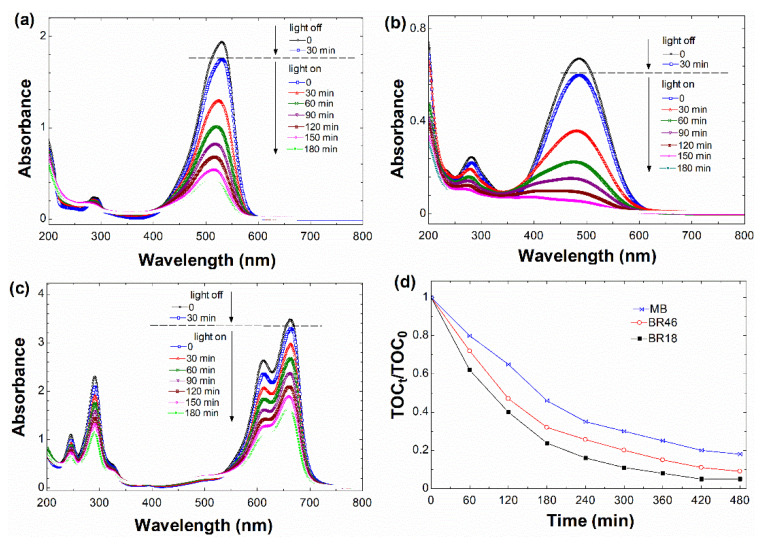
Temporal changes in absorbance spectra of dyes: (**a**) BR46, (**b**) BR18, and (**c**) MB in visible/rGO/H_2_O_2_ system, and (**d**) their corresponding TOC/TOC_0_ changes.

The UV-Vis spectral changes plots of the dyes in the visible/rGO/H_2_O_2_ system were recorded under optimum conditions with relation to time (Figure 8). The dyes’ absorption maxima were found to decline progressively, concurrently with the loss of color intensity, showing that the dyes’ chromophore was the most active location for oxidation assault. The outcomes of the TOC measurements taken throughout the entire oxidation process are shown in Figure 8d. After 480 min of metal-free Fenton process, the TOC data revealed that BR46 and BR18 had nearly mineralized into CO_2_ and H_2_O. A similar trend was observed in the case of MB in the lower extend.

### 3.3. Recyclability and Stability

**Figure 9:**
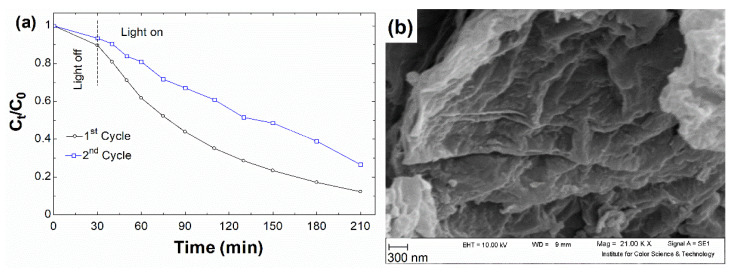
(**a**) Plots of cycling photodegradation of BR46 by rGO at the optimum condition (**b**) The microscopic image of recycled rGO after the first cycle photodegradation of BR46.

Consecutive cycle experiments were used to evaluate the rGO’s recyclability and stability, and the results are given in Figure 9a. When compared to the first run’s adsorption performance, the recycled rGO showed a modest decrease in inactivity, which might be attributed to adsorption intimidated by by-products of degraded BR46 on the rGO’s active surface. When the light was turned on, a slight drop in the catalytic performance was seen in relation to the fires cycle, presumably due to loss of catalyst and change in its structure in the presence of HO^•^. The susceptibility of the graphene sheets could explain the latter reason for oxidative attack from HO^•^ [88,89]. The morphology of the recycled catalyst was probed in order to support this hypothesis (Figure 9b). As can be seen, the recycled rGO showed no notable modifications, suggesting the chromophore of BR46 is the most preferred site for HO^•^ attack.

### 3.4. Efficiency Comparison of the Metal-Free Catalytic Activity of Graphene Derivatives Reported in the Literature

Table 2 presents a comparison of the metal-free catalytic activity of rGO (this work) with other rGOs in the literature. As mentioned earlier, rGO was used as the metal-free catalyst to promote Fenton-like degradation of textile dyes with 88%, 92%, and 70% efficacy for BR46, BR18, and MB, respectively. Compared to other studies listed in Table 2, our study had the advantage of using the lowest additives both in the case of rGO dosage and H_2_O_2_. Hence, the activity of rGO could be considered a promising metal-free catalyst material because of its photodegradation performance in visible light.

## 4. Conclusions

In this paper, rGO was synthesized and used as a visible-light-driven carbocatalyst for photocatalytic and metal-free Fenton processes. The rGO was characterized using XRD, TGA, SEM, FTIR, UV-vis, and TEM. The degradation of three textile dyes (BR46, BR18, and MB) under visible light irradiation was found to be increased by increasing catalyst dosage and decreased by increasing dye concentration. Decolorization kinetics was found to be described well by the pseudo-first-order kinetic model. The absorption maxima of the dyes decreased during the reaction, indicating the susceptibility of the chromophore of the dyes. The recycled rGO did not show any changes, signifying the recyclability of rGO during metal-free Fenton catalysis. All these advantages suggest that rGO could be recommended for use as a suitable metal-free catalyst in order to promote Fenton reaction for degradation of textile dyes.

## Figures and Tables

**Table 1:** The obtained pseudo-first-order kinetic constants for the degradation of BR46, BR18, and MB in different conditions.

Parameters	BR46		BR18		MB	
	k (min^−1^)	R^2^	k (min^−1^)	R^2^	k (min^−1^)	R^2^
	**Catalyst dosage (g/L)**					
Without catalyst	0.0046	0.9861	0.0024	0.8863	0.0006	0.9026
0.01	0.0061	0.9963	0.0030	0.8602	0.0007	0.8999
0.02	0.0071	0.9861	0.0059	0.8851	0.0010	0.9018
0.04	0.0065	0.9885	0.0048	0.8688	0.0013	0.9759
	**H_2_O_2_ addition (mM)**					
0.04 without catalyst	0.0081	0.8804	0.0085	0.9637	0.0048	0.9903
0.04 at optimum catalyst dosage	0.0112	0.9982	0.0120	0.9825	0.0063	0.9679
	**Dye (mg/L)**					
20	0.0112	0.9982	0.0120	0.9825	0.0063	0.9679
30	0.0072	0.9605	0.0037	0.9132	0.0043	0.9915
40	0.0046	0.8952	0.0023	0.9906	0.0030	0.9751
50	0.0030	0.8789	0.0013	0.9802	0.0014	0.9757
	**pH**					
3.0	0.0082	0.9967	0.0077	0.9483	0.0049	0.9878
4.5	0.0112	0.9982	0.0120	0.9825	0.0063	0.9679
6.0	0.0104	0.9862	0.0104	0.9727	0.0058	0.9746
7.0	0.0093	0.9855	0.0082	0.9251	0.0051	0.9881

**Table 2:** A summarized list of the investigation reported a metal-free catalytic activity of graphene and its derivatives materials for Fenton-like degradation of pollutants.

Sample	Method of Synthesis	Target Pollution and Concentration	Light Source	H_2_O_2_ (mM)	rGO Dosage (g/L)	pH	Time	Efficiency (%)	Ref.
rGO	Oxidation by modified Hummers’ method following with thermal reduction at 200 °C	Phenol (100 mg/L)	Visible	5.88	0.2	3	150 h	≈90	[28,29]
rGO	Oxidation by modified Hummers’ method following with thermal reduction at 350 °C	Bisphenol A (10 mg/L)	No light	10	0.4	6.5	120 min	20	[90]
		2 chlorophenol (10 mg/L)						≈40	
rGO	Oxidation by modified Hummers’ method following with solvothermal reduction at 160 °C	Methylene blue (50 mg/L)	UV-C (light intensity: 60 W m^−2^)	5.88	0.02	11	6 h	≈90	[91]
rGO	Oxidation by modified Hummers’ method and two-step reduction via hydrothermal and calcination in N_2_ atmosphere	Basic red 46 (20 mg/L)	Visible	0.04	0.04	4.5	210 min	≈88	Present study
		Basic red 18 (20 mg/L)						≈92	
		Methylene blue (20 mg/L)						≈70	

## Data Availability

Not applicable.
